# Blood glucose concentration and risk of liver cancer: systematic review and meta-analysis of prospective studies

**DOI:** 10.18632/oncotarget.16816

**Published:** 2017-04-04

**Authors:** Hedong Han, Tianyi Zhang, Zhichao Jin, Honglei Guo, Xin Wei, Yuzhou Liu, Qi Chen, Jia He

**Affiliations:** ^1^ Department of Health Statistics, Second Military Medical University, Shanghai 200433, China; ^2^ Department of Gastroenterology, Changhai Hospital, Second Military Medical University, Shanghai 200433, China; ^3^ Mount Sinai St. Luke's and West Medical Center, New York, NY 10025, USA

**Keywords:** blood glucose, liver cancer, meta-analysis, prospective studies

## Abstract

The question of whether elevated blood glucose is a risk factor for liver cancer has been intensively studied, yet with inconsistent results. To explore the relationship between blood glucose concentration and risk of liver cancer, we conduct a meta-analysis of prospective studies. Literature search was comprehensively performed using database of PubMed, EMBASE and the Cochrane Library through October 2016. Random-effect models were used to combine the effect estimations. Eight articles containing ten studies with a total of 1975 liver cancer cases were included. The pooled RRs demonstrated that elevated fasting blood glucose was associated with increased risk of liver cancer (combined RRs: 1.77; 95% CI: 1.46, 2.13) with mild heterogeneity (I^2^ = 30.40%, *P* = 0.17). In sensitivity analysis, the pooled result remained significant (combined RRs: 1.33; 95% CI: 1.12, 1.59; I^2^ = 33.90%, *P* = 0.16) when we restricted blood glucose categories in the range of nondiabetic subjects. We also detected a J-shaped non-linear dose-response relationship between blood glucose concentration and risk of liver cancer. There is evidence that elevated blood glucose increases risk of liver cancer across the range of prediabetes and diabetes. Considering the rapidly increasing prevalence of prediabetes and diabetes, controlling blood glucose may lower the risk of liver cancer.

## INTRODUCTION

Liver cancer is the six most commonly diagnosed cancer and the leading cause of gastrointestinal cancer-related death worldwide, especially in China [1, 2]. The distribution of liver cancer incidence rate is regional or country-specific, with observably higher incidence rate in developing countries. Although the incidence rate in developed countries is lower compared to that in high-risk areas, it has increased in many western countries in recent years, such as North America and most European countries [3]. It's worth noting that a striking gender disparity in liver cancer incidence has been well described, with 2–3 fold rates in males than females [4, 5]. Moreover, males with liver cancer tend to have a worse prognosis and a higher risk of recurrence [6, 7]. Considering the world's liver cancer burden and preventable characteristic of this disease, etiologic studies are required to elucidate more risk factors and consequently to prevent liver cancer incidence.

A vast number of factors have been recognized to contribute to the incidence, progression and outcome of liver cancer. Interestingly, these identified risk factors also vary between high-rate and low-rate regions [8]. Chronic hepatitis B virus (HBV) infection and aflatoxin B_1_ exposure play dominant roles in most high-rate countries, however, in low-rate countries, obesity, overdrinking, hepatitis B virus (HCV) infection and metabolic syndrome are the major risk factors. Diabetes was reported to associate with a significant 101% increase in risk of liver cancer based on a previous meta-analysis of twenty-five cohort studies [9].

Hepatocarcinogenesis caused by hyperglycaemia and hyperinsulinemia through upregulating the production and bioavailability of insulin-like growth factor-1 (IGF-1) has been observed in animal models, *in vitro* studies and epidemiological studies [10, 11]. However, as an indication of elevated blood glucose, hyperglycaemia may appear at the stage of prediabetes (including impaired fasting glycaemia and/or impaired glucose tolerance), which is defined with a range of blood glucose between upper normal limit and diabetes (fasting blood glucose 100–125 mg/dl) [12]. This provides the possibility that prediabetes may also increase the risk of liver cancer.

The previous epidemiological studies referring to the association between blood glucose concentration and liver cancer risk yielded conflicting results [13–20]. Particularly, some studies reported a significant association between prediabetes and liver cancer risk [13, 19, 20], however, others found null association [14–16, 18]. Prediabetes has important implications for prevention in clinical practice, as interventions in lifestyle like physical activity, weight change or modification of diet could improve or even reverse the status [21, 22]. Therefore, we performed a meta-analysis of prospective studies for the purposes as follows: 1) to summarize all the available prospective evidence to evaluate the association between fasting blood glucose and liver cancer risk; 2) to investigate whether prediabetes is a risk factor for liver cancer or not; 3) to explore the potential linear and non-linear dose-response relationship between fasting blood glucose and risk of liver cancer.

## MATERIALS AND METHODS

### Search strategy

The review was registered in PROSPERO-international prospective register of systematic reviews (registration number. CRD42016049496). We designed, analyzed and reported this meta-analysis following the Meta-analysis of Observational Studies in Epidemiology (MOOSE) guidelines [23]. We searched electronic databases of PubMed, EMBASE and the Cochrane Library for potential prospective studies published before October 2016. The following terms were used for searching: (‘liver cancer’ OR ‘hepatocellular carcinoma’ OR ‘hepatic carcinoma’ OR ‘liver tumors’ OR ‘liver neoplasms’) AND (‘serum glucose’ OR ‘blood glucose’ OR ‘fasting glucose’ OR ‘plasma glucose’). No language restrictions were imposed. The reference lists of included articles were reviewed for additional studies.

### Study selection

Studies were included in this meta-analysis if they met the following criteria: 1) had a prospective design (cohort or nested case-control); 2) reported measurements of blood glucose levels; 3) the outcome of interest was liver cancer incidence; 4) adjusted RRs and corresponding 95% confidence intervals (CIs) were provided.

### Data extraction and quality assessment

A predefined data extraction form was applied to extract data from relevant articles by two reviews (H.H. and T.Z.) independently. The following information was extracted: the first author's last name, year of publication, study location, study design, age, gender, baseline, number of cases, follow-up time, contrast of the most extreme quantiles, estimated effect size, corresponding 95% CI and adjusted factors. Quality assessment was conducted using Newcastle-Ottawa Quality Assessment Scale (NOS) and studies with an NOS score ≥7 were considered high quality [24].

### Statistical analysis

We calculated the summary RRs of liver cancer for the highest versus lowest level of blood glucose using the random-effect model described by DerSimonian and Laird, which is in consideration of heterogeneity both between and within studies [25]. If study reported RRs for different genders (men and women) separately, yet without the overall results, then the result of each gender was regard as a different study. Fasting blood glucose is regarded as the exposure because it is the most frequently used criteria for diagnosis of prediabetes and diabetes [26]. We transformed relevant data using method described by Liao et al. [27] for the study that determined serum glucose levels undergoing a 1-h glucose tolerance test [14]. Forest plots were used to display the RRs and corresponding 95% CI. We further used I^2^ statistics to assess heterogeneity across the studies [28]. Publication bias was assessed with the Egger's test and the funnel plot [29].

For the dose-response analysis, we used the method proposed by Greenland et al., Longnecker et al. [30] and Orsini et al [31] to estimate the linear trends of RR for liver cancer per 0.56 mmol/L (10 mg/dL) increase in fasting blood glucose from the correlated estimations across categories of blood glucose. In addition, to examine a potential non-linear association between blood glucose and risk of liver cancer, we conducted a two-stage random-effect dose–response meta-analysis using restricted cubic splines with three knots at fixed percentiles (10%, 50% and 90%) of the distribution [31, 32]. A *P*-value for non-linearity was calculated by testing the hypothesis that the coefficient of the second spline was different from zero. We assigned the dose in each category using the midpoint or median level of blood glucose. However, if lower boundary of the lowest category was not reported, it was assumed as 3.9 mmol/L (70.2 mg/dL) for the reason that the lower limit of normal fasting glucose was approximately 3.9 mmol/L [33]. If the highest category was open-ended, the higher boundary was calculated as the lower bound plus 1.5 times the width of the closest category.

Finally, we first performed sensitivity analysis by omitting any specific study to assess the robustness of the pooled results (leave-one-out sensitivity analysis). Secondly, we repeated the analysis by restricting blood glucose categories in the range of nondiabetic subjects (levels below 126 mg/dl) to detect whether the increased risk of liver cancer was caused by diabetes. Subgroup analysis was conducted based on study region, gender, category number, number of cases, follow-up time and control for potential confounders. Stata Version 12.0 software (Stata Corp, College Station, TX) was used for all analyses, and *P*-value < 0.05 was considered to be statistically significant.

## RESULTS

### Study characteristics

**Figure 1:**
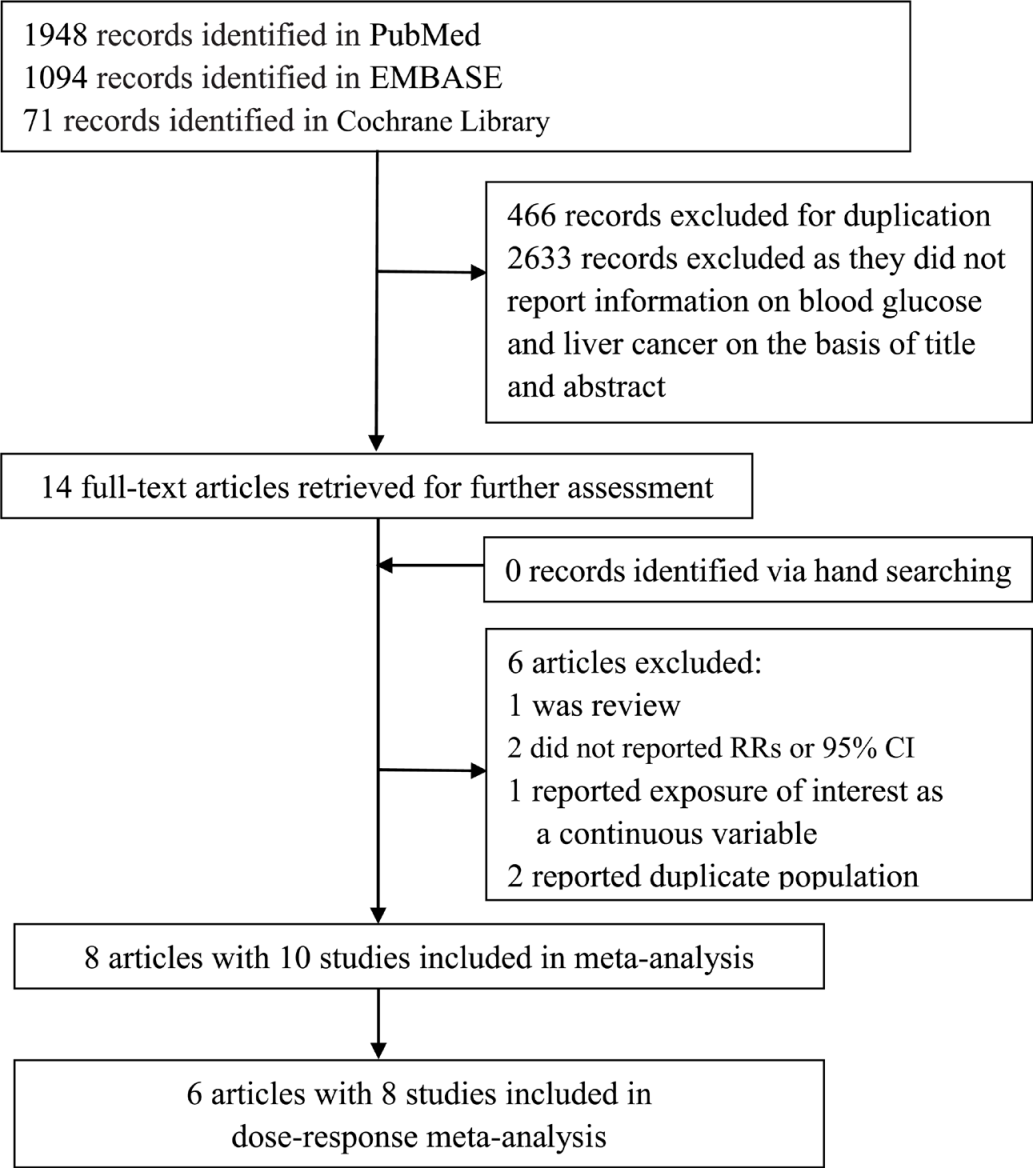
Flow chart of literature search for studies investigating association between fasting blood glucose and risk of liver cancer

We identified 3113 studies through a comprehensive search and 3099 studies were eliminated after review of title or abstract (Figure 1). In the assessment of 14 full-text articles, 6 studies were further excluded for the following reasons: 2 studies reported duplicate population; 1 was review; 2 did not provide RR or 95% CI; 1 reported blood glucose as continues variable. Finally, 8 articles containing 10 studies with a total of 1975 liver cancer cases were included in this meta-analysis. The characteristics of the included studies were summarized in Table 1. 4 studies (1 in US, 1 in Finland and 2 in Austria) were conducted in low-risk region of liver cancer and 6 studies (1 in China, 2 in Japan and 3 in Korea) were conducted in high-risk region. Considering gender distribution, 4 studies reported both genders (men and women), 5 reported men and 2 reported women. The study conducted by Rapp et al. [19] reported the combined result and result for men, but did not provide the result for women. The mean follow-up time among the included studies ranged from 2 to 22.5 years and 8 were more than 10 years. 3 studies were adjusted for HBV or HCV infection and most were adjusted for BMI, smoking and alcohol consumption. All the included studies had a relatively high-quality (NOS score ≥ 7).

**Table 1:** Baseline characteristics of all the studies included in the meta-analysis

First Author, Year, Rigion	Study design	Age (mean years)	No. of Cases	Gender (women %)	Baseline	Follow-up (mean years)	Contrast of most extreme quantiles (mg/dl)	RR (95% CI) (Highest vs. lowest)	Adjustments	NOS
Loftfield, 2016, Finland	Nested case-control	50–69	138	0	1985–1988	22	110–124 versus 86–92	2.40 (1.33–4.35)	Age, cigarettes per day, duration of smoking, alcohol intake, anti-HBc, HBsAg, anti-HCV, BMI, education.	7
Petrick, 2016, USA	Nested case-control	Cases:41.6 Control:44.4	450	Both (32.7)	1964–1992	22,5	≥ 126 versus < 100	1.63 (0.48,1.55)	Period of enrollment, age, date of blood draw, sex, race, BMI, diabetes.	8
Borena, 2012, Austria	Cohort	44	266	Both (49.9)	1972–2005	12	Mean 120.6 versus Mean 73.8	2.78 (0.78,9.96)	Age, sex, smoking status, cohort, categories of birth year, BMI.	8
Chao, 2011, China	Cohort	30–65	124	0	1989–2006	17	≥ 126 versus < 110	2.37 (1.12, 5.04)	Age, number of visits, smoking, alcohol consumption, a first-degree family history of HCC, baseline viral Factors.	8
Inoue, 2009, Japan	Cohort	40–69	74	0	1993–1995	10.2	≥ 100 versus < 100	1.76 (1.07, 2.89)	Age, study area, smoking status, weekly ethanol intake, total serum cholesterol.	8
Inoue, 2009, Japan	Cohort	40–69	40	100	1993–1995	10.2	≥ 100 versus < 100	1.18 (0.94,2.86)	Age, study area, smoking status, weekly ethanol intake, total serum cholesterol.	8
Gwack, 2007, Korea	Cohort	56.8	36	61.8	1993–2004	3.4	≥ 126 versus < 100	2.77 (1.24, 6.18)	Age, gender, smoking, alcohol consumption, education level, BMI, hepatitis B antigen seropositivity.	7
Rapp, 2006, Austria	Cohort	43	49	55.2	1988–2001	8.4	≥ 133.2 versus [75.6–93.6]	4.58 (1.81,11.62)	Age, smoking status, occupational group, BMI.	8
Jee, 2005, Korea	Cohort	30–95	700	0	1992–1995	10	≥ 140 versus < 90	1.72 (1.56, 1.89)	Age, age squared, amount of smoking, alcohol use.	8
Jee, 2005, Korea	Cohort	20–79	98	100	1992–1995	10	≥ 140 versus < 90	1.22 (0.91, 1.63)	Age, age squared, amount of smoking, alcohol use.	8

Abbreviations: RR, relative risk; CI, confidence interval; NOS, Newcastle–Ottawa Quality Assessment Scale; BMI, body mass index; HCC, hepatocellular carcinoma; Anti-HBc, antibodies to hepatitis B core antigen; HBsAg, hepatitis B surface antigen; Anti-HCV, anti-hepatitis C virus.

### Fasting blood glucose and risk of liver cancer

**Figure 2:**
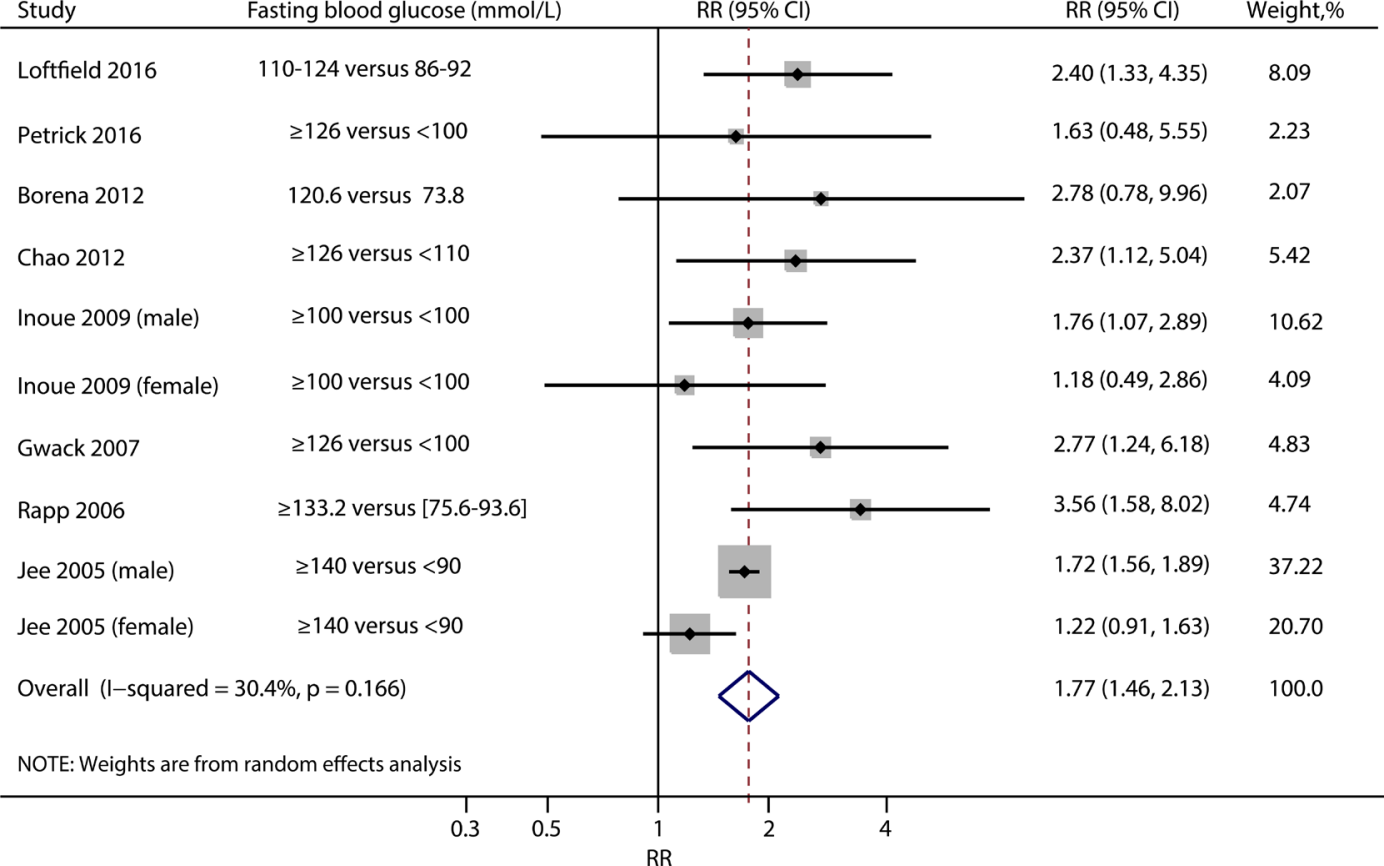
Summary risk ratios of liver cancer for the highest compared to the lowest categories of fasting blood glucose

**Figure 3:**
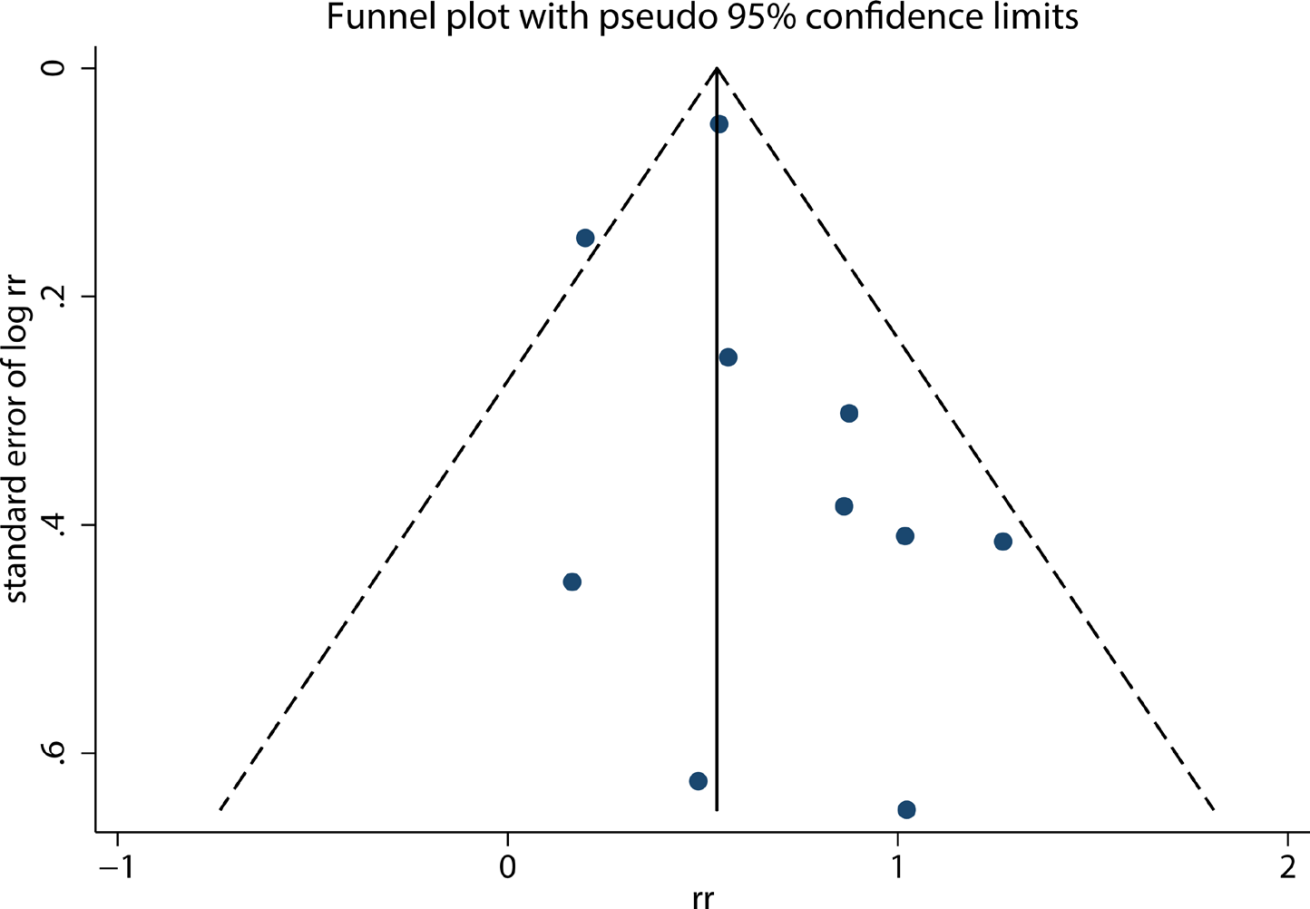
Funnel plot of studies reporting fasting blood glucose and liver cancer risk

The summary RRs of liver cancer risk for the highest vs. the lowest categories of fasting blood glucose were shown in Figure 2. The pooled RR was 1.77 (95% CI: 1.46, 2.13) with mild heterogeneity among the included studies (I^2^ = 30.40%, *P* = 0.17). No evidence of publication bias was detected (Egger test, *P* = 0.37; Funnel plot, Figure 3).

### Dose-response analysis

For the dose-response analysis, we excluded study conducted by Inoue et al. that only reported two categories because at least three categories were needed for the study-specific trend estimation. The pooled RRs for risk of liver cancer were 1.11 (95% CI: 1.06, 1.17; I^2^ = 78.30%, *P* < 0.001) for per 0.56 mmol/L (10 mg/dL) increase in fasting blood glucose.

**Figure 4:**
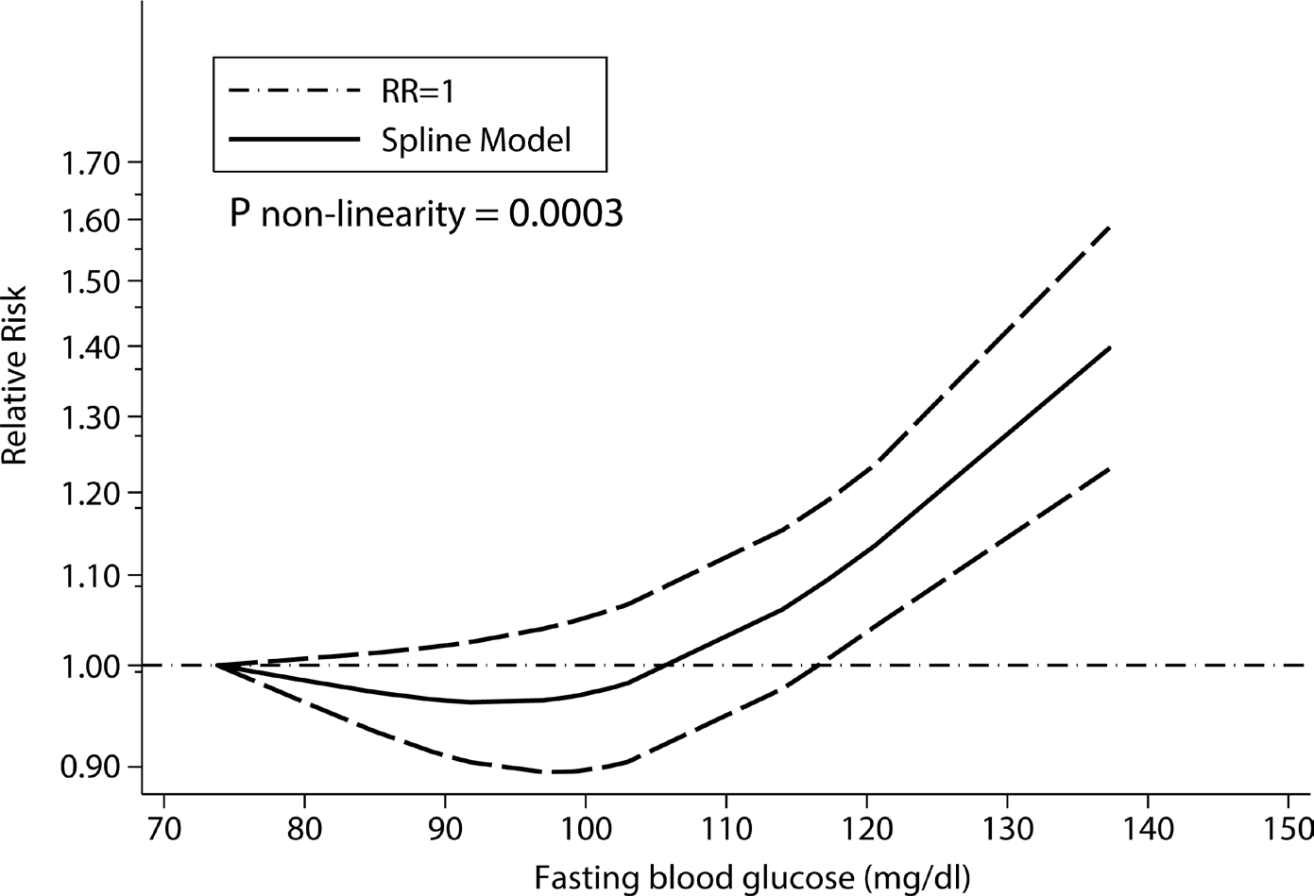
Dose–response relationship between fasting blood glucose and liver cancer risk

According to Figure 4, there was a J-shaped non-linear relationship between fasting blood glucose and risk of liver cancer (P for non-linearity < 0.001). The pooled RRs of liver cancer risk calculated from the spline model began to be significant as blood glucose concentration increased to approximately 6.50 mmol/L (117 mg/dL).

### Subgroup and sensitivity analysis

The results of subgroup analysis were shown in Table 2. When stratified by study region, category number, number of cases, follow-up time and several adjustments, our results remained significant. The combined RRs were 2.59 (95% CI: 1.70, 3.94) in low-risk region and 1.63 (95% CI: 1.33, 1.99) in high-risk region. After adjusted for HBV or HCV infection, the pooled RRs were 2.48 (95% CI: 1.66, 3.71). For gender disparity, higher blood glucose increased liver cancer risk in male participants (combined RRs: 1.98; 95% CI: 1.55, 2.53) but not in female participants (combined RRs: 1.22; 95% CI: 0.92, 1.60).

**Table 2:** Subgroup analysis of blood glucose concentration and risk of liver cancer

Subgroup	Studies, *n*	Cases	RR (95% CI)	I^2^ (%)	*P*
Total	10	1975	1.77 (1.46, 2.13)	30.40	0.17
Study region					
Low-risk	4	903	2.59 (1.70, 3.94)	0	0.75
High-risk	6	1072	1.63 (1.33, 1.99)	35.90	0.17
Gender					
Both	4	801	2.79 (1.73, 4.50)	0	0.78
Men	5	1036	1.98 (1.55, 2.53)	32.70	0.20
Women	2	138	1.22 (0.92, 1.60)	0	0.94
Category number					
= 2	2	114	1.60 (1.04, 2.46)	0	0.44
≥ 3	8	1861	1.85 (1.47, 2.34)	42.80	0.09
No. of cases					
< 100	5	297	1.77 (1.18, 2.65)	15.70	0.31
≥ 100	5	1678	1.75 (1.59, 1.92)	0	0.67
Follow-up time (years)					
< 10	2	85	3.14 (1.77, 5.55)	42.90	0.15
≥ 10	8	1890	1.66 (1.42, 1.93)	0	0.92
Adjustments					
HBV or HCV infection					
Yes	3	298	2.48 (1.66, 3.71)	0	0.95
No	7	1667	1.64 (1.32, 2.03)	36.20	0.15
BMI					
Yes	5	939	2.63 (1.81, 3.81)	0	0.87
No	5	1036	1.58 (1.30, 1.93)	36.40	0.18
Smoking					
Yes	8	1387	1.74 (1.40, 2.16)	39.80	0.11
No	2	588	2.23 (1.31, 3.80)	0	0.58
Alcohol consumption					
Yes	7	1210	1.69 (1.39, 2.04)	34.60	0.16
No	3	765	2.80 (1.54, 5.09)	0	0.58

Abbreviations: RR, relative risk; CI, confidence interval; BMI, body mass index; HBV, hepatitis B Virus; HCV, hepatitis C virus.

Sensitivity analysis in our study indicated similar results. The summary RRs of liver cancer risk for the highest vs. the lowest categories of fasting blood glucose remained significant (combined RRs: 1.19; 95% CI: 1.10, 1.28) when we restricted blood glucose categories in the range of nondiabetic subjects (levels below 126 mg/dl). The *P*-value for non-linear relationship between fasting blood glucose and risk of liver cancer also remained significant (*P* = 0.0021) after we restricted blood glucose categories in the range of nondiabetic subjects (levels below 126 mg/dl).

## DISCUSSION

To the best of our knowledge, this is so far the first meta-analysis to summarize evidence on the relationship between fasting blood glucose and risk of liver cancer. According to the results of our study, elevated fasting blood glucose was significantly associated with increased risk of liver cancer across the range of prediabetes and diabetes. The risk of liver cancer quantitatively increased by 77% in the category of highest blood glucose compared with the lowest. We further found a J-shaped non-linear relationship between fasting blood glucose and risk of liver cancer.

As shown by the results of subgroup analyses, the risk of liver cancer increased with the rises in blood glucose concentration in all subgroups except for subgroup that was stratified by gender. Null association was found in female subjects in this study. The gender disparity in liver cancer incidence has been long well described and it may attribute to a higher prevalence of smoking, alcohol consumption, HBV or HCV infection in males. Our results revealed that susceptibility to higher blood glucose in males may also contribute to the gender disparity of liver cancer occurrence. However, because of a limited studies reporting the result and potential lack of statistical power, this result should be cautiously interpreted. Considering study region, the significant results remained stable no matter in high-risk or low-risk areas. Several risk factors have been established for liver cancer, including HBV or HCV infection [34], obesity [35], smoking [36] and alcohol consumption [37]. When we restricted studies that were adjusted for these factors, the results of subgroup analyses did not alter substantially. Therefore, our subgroup analyses indicated that elevated blood glucose was a modifiable risk factor for liver cancer independently of above-mentioned confounders.

Diabetes (defined as FBG >126 mg/dl) was associated with increased risk of hepatocellular carcinoma in both males and females based on a latest meta-analysis of 25 cohort studies. However, the role of prediabetes (defined as FBG 100–125 mg/dl) on incidence of liver cancer risk was still uncertain. Significant association between prediabetes and liver cancer risk was found in three previous studies and null association was found in five studies. In the sensitivity analysis, when the blood glucose categories above 126 mg/dl were excluded, significant associations for elevated blood glucose and non-linear dose-response relationship still existed, which confirmed the hypothesis that prediabetes status increase liver cancer risk.

Several possible mechanisms have been proposed to explain the hypothesis between hyperglycaemia and increased liver cancer incidence. Experimental and epidemiological studies have suggested that hyperglycaemia and hyperinsulinemia may upregulate the production and bioavailability of insulin-like growth factor-1 (IGF-1), which could promote cellular proliferation and inhibits apoptosis by receptor-mediated pathways in live cancer cells [10, 38, 39]. In addition, subjects with elevated blood glucose were generally companied with insulin resistance, which may result in higher level of pro-inflammatory cytokines, such as tumor necrosis factor-alpha (TNF-α) and interleukin-6 (IL-6). These pro-inflammatory factors have been proved to cause chronic inflammation that would subsequently promote hepatocarcinogenesis [40]. As a result of overburden on glucose oxidation, hyperglycemia can add oxidative stress in hepatic cell with the generation of reactive oxygen species (ROS), which can bind DNA, cause genetic variation and finally promote the occurrence of liver cancer [41–43].

Important strengths should be acknowledged in this meta-analysis. All studies met the inclusion criteria had a prospective design. As liver cancer itself would induce diabetes or hyperglycemia, concerns might raise that the association between elevated blood glucose and liver cancer could be partly caused by reverse causality. Under such assumption, there might be a positive feedback loop in exploring the cause-effect relationship between blood glucose concentration and risk of liver cancer. To exclude possible reverse causality from diabetes or hyperglycemia induced by liver cancer, we repeated the analysis by restricting blood glucose categories in the range of nondiabetic subjects and the result was robust. In addition, we used NOS score to assess the study quality and all studies had a high-quality. As the sample size of primary single study was relatively small, our study increased the statistical power to detect possible association between fasting blood glucose and liver cancer risk and further determined a precise risk estimation. Sensitivity analyses in this meta-analysis proved the stability of our conclusions concerning comparison of the highest category versus the lowest category and non-linear dose-response relationship after omitting one study in turn and restricting blood glucose categories in the range of nondiabetic subjects (levels below 126 mg/dl).

Potential limitations should be considered. First, exposure measured only at baseline may not reflect blood glucose levels over a long time follow-up. Especially for subjects with hyperglycemia or diabetes, they tended to control glucose by taking antidiabetic medications or improving life style, which couldn't be neglected as bias would be introduced under these conditions. Second, gender-specific risk for female participants was analyzed in only two studies, although both studies indicated null association. This phenomenon should be further examined and confirmed. Third, in spite of some important confounders seemed unlikely to alter the role of fasting blood glucose on the increment of liver cancer risk based on our subgroup analyses of adjustments, other factors potentially accounted for the observed association cannot be ruled out. For example, information with respect to use of antidiabetic medications was not available in most studies. Four, With regards to subtypes of liver cancer such as hepatocellular carcinoma (the most common type), hepatoblastoma, cholangiocarcinoma and other rare ones, the included studies did not provide corresponding effect estimations, respectively. Further studies should place more emphasis on association between blood glucose concentration and less common subtypes of liver cancer and help to identify more risk factors for these kinds of liver cancer.

### In conclusion

This meta-analysis provides evidence for the hypothesis that fasting blood glucose is significantly associated with increased risk of liver cancer, and this association is dose-dependent. The combined result of increased liver cancer risk begins to be significant as blood glucose concentration is above approximately 6.50 mmol/L (117 mg/dL). Considering the rapidly increasing prevalence of prediabetes and diabetes, controlling blood glucose may lower the risk of liver cancer.
